# Effect of low-level laser therapy (808 nm) on skeletal muscle after endurance exercise training in rats

**DOI:** 10.1590/bjpt-rbf.2014.0113

**Published:** 2015-09-01

**Authors:** Livia Assis, Fernanda Yamashita, Angela M. P. Magri, Kelly R. Fernandes, Liria Yamauchi, Ana C. M. Renno

**Affiliations:** 1 Departamento de Biociências, Universidade Federal de São Paulo (UNIFESP), Santos, SP, Brazil; 2 Departamento de Ciências do Movimento Humano, Universidade Federal de São Paulo (UNIFESP), Santos, SP, Brazil

**Keywords:** low-level laser therapy, endurance exercise, lactate, skeletal muscle, myogenin, physical therapy

## Abstract

**BACKGROUND:**

Low-level laser therapy (LLLT) has been demonstrated to be effective in optimizing skeletal muscle performance in animal experiments and in clinical trials. However, little is known about the effects of LLLT on muscle recovery after endurance training.

**OBJECTIVE:**

This study evaluates the effects of low-level laser therapy (LLLT) applied after an endurance training protocol on biochemical markers and morphology of skeletal muscle in rats.

**METHOD:**

Wistar rats were divided into control group (CG), trained group (TG), and trained and laser irradiated group (TLG). The endurance training was performed on a treadmill, 1 h/day, 5 days/wk, for 8 wk at 60% of the maximal speed reached during the maximal effort test (Tmax) and laser irradiation was applied after training.

**RESULTS:**

Both trained groups showed significant increase in speed compared to the CG. The TLG demonstrated a significantly reduced lactate level, increased tibialis anterior (TA) fiber cross-section area, and decreased TA fiber density. Myogenin expression was higher in soleus and TA muscles in both trained groups. In addition, LLLT produced myogenin downregulation in the TA muscle of trained animals.

**CONCLUSION:**

These results suggest that LLLT could be an effective therapeutic approach for stimulating recovery during an endurance exercise protocol.

## Introduction

Low-level laser therapy (LLLT) is an innovative clinical approach commonly used to treat inflammatory processes, pain, and muscle skeletal tissue injury^1^^-^ 3. This technology has recently showed a positive effect on the stimulation of cell activities involved in the healing process^4^^,^ 5.

LLLT acts on the cell's bioenergy, increasing the availability of cellular energy^6^^-^ 10. Some studies have demonstrated that LLLT is able to produce structural and metabolic changes in the organelles of different cells and/or tissues, including the formation of giant mitochondria, which may provide higher levels of respiration and energy (ATP) to cells^7^. Moreover, recent systematic reviews demonstrated that LLLT attenuates the muscle's inflammatory mediators and enhances activity of antioxidant enzymes when applied after or before exercise^11^^-^ 13. Thus, these physiological adaptations could improve muscular performance and decrease fatigue during physical exercise programs^7^^,^ 14 ^,^ 15.

In this context, some authors demonstrated positive effects of LLLT using experimental and clinical models of fatigue^14^^-^ 16. Sussai et al.^16^ observed that LLLT (660 nm, 100 mW, 133.3 J/cm^2^) decreased serum levels of creatine kinase (CK) after a swimming-induced muscle fatigue protocol in rats. Similar results were found by Almeida et al.^17^ using 808 nm laser and by Leal et al.^10^ using 904 nm laser. The latter demonstrated a decrease in the skeletal muscle damage related to the exercise and a delay in muscle fatigue, enhancing skeletal muscle performance in both healthy volunteers and athletes. Moreover, dos Reis et al.^15^ demonstrated that LLLT either before or after fatigue protocol reduced the concentrations of serum lactate and CK in male soccer players.

Other authors also investigated the effects of LLLT associated with exercise training. Vieira et al.^6^ observed that LLLT combined with an endurance training program produced a greater reduction in fatigue compared to endurance training only in young female volunteers. Furthermore, Ferraresi et al.^18^ showed that LLLT associated with strength training can increase muscle performance compared with strength training only.

In addition, studies using other light sources, such as light-emitting diode therapy (LEDT), have been performed for the same purpose^19^^,^ 20. The results showed that the LEDT treatment was able to reduce CK, lactate, and C-reactive in blood, and it increased the number of repetitions and time of contraction in human physical exercise^19^^,^ 20.

Despite the positive effects of LLLT on exercised muscle, most of the studies investigated the acute effect of this therapeutic approach on muscle performance or fatigue. Therefore, there is a lack of research demonstrating the effects of LLLT on chronic exercised muscles, especially in conjunction with aerobic exercise. Based on the promising effects of LLLT on cell metabolism and energy supply modulation, it was hypothesized that this therapeutic approach may favor muscular recovery, improving efficiency during a physical exercise program in rats. For these reasons, the present study aimed to evaluate the effects of 808 nm laser applied after an endurance training protocol on biochemical markers and morphology of skeletal muscle in rats.

## Method

### Experimental design

Twenty-four male Wistar rats (aged 6 weeks and body mass ± 200 g) were used in this study. They were maintained under controlled temperature (22±2^o^C), light-dark periods of 12 hours and with free access to water and commercial diet. All animal handling and procedures were strictly conducted according to the Guiding Principles for the Care and Use of Laboratory Animals. The animal experimental plan was reviewed and approved by the Animal Experimentation Ethics Committee of Universidade Federal de São Paulo/Hospital São Paulo (UNIFESP), São Paulo, SP, Brazil (CEP-0222/12), and the national guidelines for animal care were observed.

Rats were randomly distributed into three groups (n=8 each group): sedentary control group (CG); trained group (TG); and trained and laser irradiated group (TLG).

### Evaluation of the maximal physical capacity of each rat - maximal effort test (Tmax)

All groups were familiarized with a motorized treadmill at a speed of 5 m/min for 5 min/day for 1 wk before the beginning of the training protocol. After the familiarization period, rats were randomly assigned into the sedentary control group and trained groups. The physical capacity of each rat was evaluated through a maximal effort test (Tmax) on the motorized treadmill, starting at a speed of 5m/min and increasing the speed in 5 m/min at each 3-min stage. The maximal physical capacity was assumed to be the speed at which the animals stopped running spontaneously.

### Endurance training

Trained groups ran on a motorized treadmill at a speed of 60% the maximal speed reached during a maximal effort test (Tmax 1), 5 days/wk for 1 h/day for a period of 8 wks. After 4 wks of training, a new maximal effort test (Tmax 2) was applied and the speed of training was recalculated. At the end of 8 wks of training, another maximal effort test (Tmax 3) was applied to evaluate the physical capacity of the rats. CG was submitted only to the three maximal effort tests^21^.

### LLLT Protocol

Photobiostimulation was performed using a gallium-aluminum-arsenide (GaAlAs) diode laser (Photon Laser II, DMC^®^ Equipment Ltda., São Carlos, SP, Brazil), with the following parameters: continuous radiation mode; 808 nm wavelength; 30 mW power output; 47 sec irradiation time; 0.00785 cm² spot area; dose 180 J/cm^2^; irradiance 3.8 W/cm^2^; 1.4 J total energy per point/section; and 5.6 J total energy per point per lower limb and 224 J total dose delivered over the whole protocol (40 sessions). After every endurance training protocol, the laser irradiation was applied to 4 sections of both lower legs on muscles involved in running (middle region of quadriceps, gluteus maximus, TA, and gastrocnemius muscles), 8 points per section, using the punctual contact technique, and the optical fiber was positioned perpendicularly to the skin.

### Lactate evaluation

Blood samples were collected from a cut at the tip of the tail at the end of the three maximal effort tests. The sample (25 μl) was immediately transferred to test tape. The lactate concentrations were determined with a hand-held portable lactate analyzer (Accutrend Plus^®^, Roche Diagnostic, Germany).

After the last lactate evaluation, rats were sacrificed individually by carbon dioxide asphyxia, and muscles were removed for analysis.

### Histology

The specimens were fixated in 4% formaldehyde for 2 days, followed by dehydration in a graded series of ethanol and embedding in paraffin, and histological sections were prepared. Therefore, for TA and soleus, thin sections (5 µm) were prepared perpendicular to the medial-lateral drilling axis using a microtome with a diamond blade (Leica Microsystems SP 1600, Nussloch, Germany). At least three sections of each specimen were stained with H.E. stain (Merck).

### Morphometric analysis

The muscle fiber cross-section area (CSA) was assessed for each histological section under a light microscope (AxioVision 4.7, Carl Zeiss, Germany), using morphometric analysis software (AxioVision 4.7.1.0, Carl Zeiss). The fiber CSA for each muscle was obtained from digital images (40X) by measuring the area of 100 fibers located in the central region of the section. A blind procedure was used for measurements.

### Muscle fiber density

The muscle fiber density (number of fibers/mm^2^- TA and soleus muscle) was determined as described by Mandarim-de-Lacerda et al.^22^. For this purpose, two cuts chosen randomly and stained with H&E were used. A total of six photomicrographs were assessed per animal. To determine the muscle fiber density, computerized imaging equipment (AxioVision 4.7, Carl Zeiss, Oberkochen, Germany) with a 40x objective was used. Two experienced observers (LA and FY) performed the scoring in a blinded manner.

### Immunohistochemistry analysis: myogenin expression

For myogenin expression analysis, the paraffin was removed with xylene from serial sections of 5 μm. After this procedure, the sections were rehydrated in graded ethanol and pretreated in a microwave with 0.01 M citric acid buffer (pH 6) for 3 cycles of 5 min each at 850 W for antigen retrieval. Subsequently, the material was pre-incubated with 0.3% hydrogen peroxide in phosphate-buffered saline (PBS) solution for 5 min to inactivate the endogenous peroxidase and then blocked with 5% normal goat serum in PBS solution for 10 min. The specimens were incubated with anti-myogenin polyclonal primary antibody (Santa Cruz Biotechnology, USA) at a concentration of 1:200. Incubation was performed overnight at 4° C in the refrigerator and followed by two washes in PBS for 10 min. Afterwards, the sections were incubated with biotin conjugated secondary antibody anti-rabbit IgG (Vector Laboratories, Burlingame, CA, USA) at a concentration of 1:200 in PBS for 1 h. The sections were washed twice in PBS followed by the application of an avidin-biotin-peroxidase complex (Vector Laboratories) for 45 min. The bound complexes visualized by the application of a 0.05% solution of 3-3'-diaminobenzidine solution and counterstained with Harris Hematoxylin. Finally, for control analyses of the antibodies, the serial sections were treated with rabbit IgG (Vector Laboratories) at a concentration of 1:200 instead of the primary antibody. Furthermore, internal positive controls were performed with each staining bath. Digital images were captured with an optical microscope (Leica Microsystems AG, Wetzlar, Germany). Nucleus fibers marked brown were considered positive for MuRF-1 and atrogin-1 expression. Two experienced observers (LA and FY) performed the scoring in a blinded manner.

### Statistics

Data are expressed as the mean±standard error of the mean (SEM) and confidence interval (CI). The Shapiro-Wilk and Levene tests were applied to evaluate the normality and homogeneity of the results, respectively. Comparisons between experimental groups were performed by analysis of variance (one-way ANOVA), and Tukey's post-hoc test was used to compare individual groups. A *P*value <0.05 was considered significant. All analyzes were performed using a GraphPad Prism 6 (GraphPad Software, San Diego CA, USA).

## Results

### Endurance training

**Figure 1.**
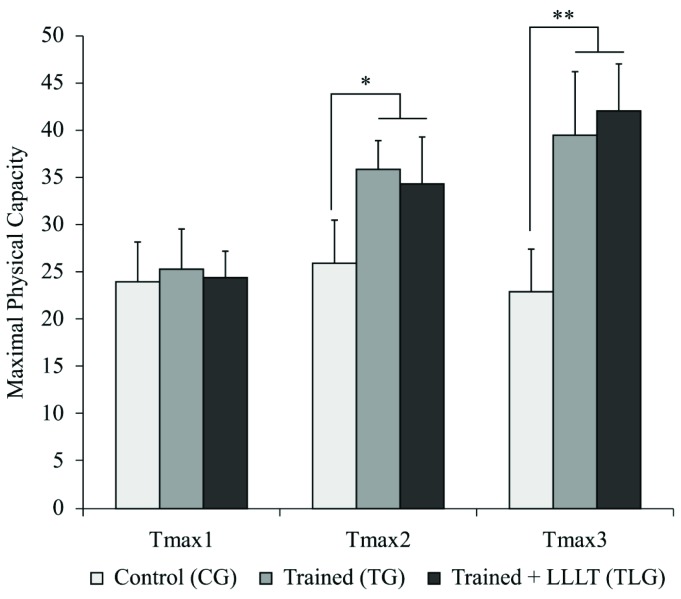
Maximal physical capacity (Tmax). CG: control group; TG: trained group; TLG: trained and laser group. (Mean±SD). *p= 0.0005 (TG) and p=0.0024 (TLG) vs CG (Tmax2); **P<0.0001 vs CG (Tmax2).

Figure 1 shows the values (speed m/min) obtained in the Tmax for all experimental groups. The values found in Tmax1 were similar for all animals. After 4 weeks of training, both trained groups presented significantly higher Tmax 2 compared to the CG [TG (p=0.0005, CI=-14.88 to -4.230), TLG (p=0.0024, CI=-13.86 to -2.885)]. However, no difference in speed was found between the TG and TLG. The same result was observed at the end of the experiment (Tmax 3), with a significant difference between the CG and trained groups [TG (p<0.0001, CI=-22.38 to -10.37; TLG p<0.0001, CI=-25.35 to -12.95)].

### Lactate evaluation

**Figure 2.**
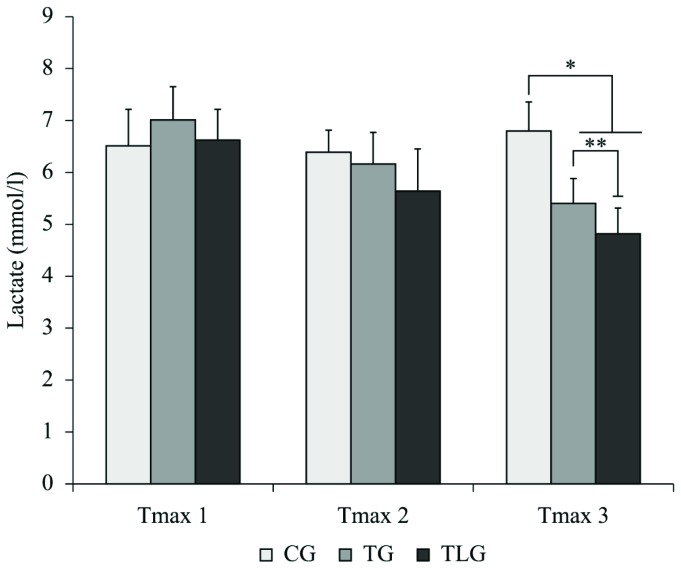
Lactate concentration. CG: control group; TG: trained group; TLG: trained and laser group. (Mean±SD). *p=0.004 vs TG **P<0.0001 vs TLG.

Similar blood lactate concentration was observed for all experimental groups after the Tmax 1 (GC, TG and TLG) and Tmax 2 (GC, TG and TLG). At the end of the experiment (Tmax 3), the lactate concentration was significantly higher in the CG compared to the TG (p=0.004, CI=0.4129 to 2.143) and TLG (p<0.0001, CI=1.211 to 3.089). Furthermore, the TLG showed a significantly lower value of lactate levels compared to the TG (p=0.035, CI=0.05501 to 1.689; Figure 2).

### Muscle fiber CSA

**Figure 3.**
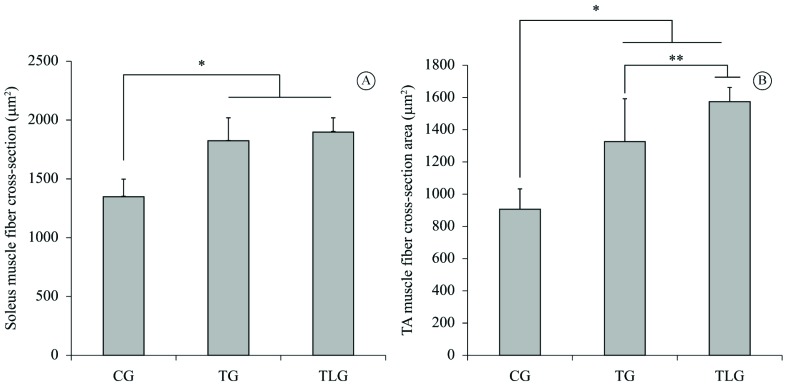
Morphometry of CSA. (A) Soleus muscle fiber CSA. CG: control group; TG: trained group; TLG: trained and laser group. (Mean±SD) *P<0.0001 (TG) and P<0.0001 (TLG) vs CG; (B) TA muscle fiber CSA * p=0.0005 (TG) and P<0.0001 (TLG) vs CG; **p=0.028 vs TG. (Mean±SD).

Morphometric analysis of muscle fiber CSA revealed that the endurance training protocol produced a significant increase in the soleus [TG (p<0.0001, CI=-671.0 to -275.7); TLG (p<0.0001, CI=-756.6 to -350.5)] and TA [TG (p=0.0005, CI=-652.6 to -179.2); TLG (p<0.0001, CI=-943.4 to -441.3) fiber CSA compared to the CG (Figure 3A and B). Furthermore, the TLG showed a significant increase in TA fiber CSA compared to the TG (p=0.028, CI=-527.5 to -25.36; Figure 3B).

### Muscle fiber density

**Figure 4.**
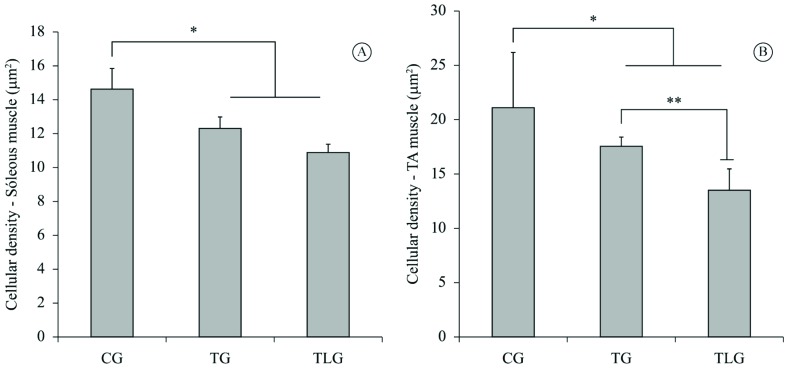
Cellular Density. (A) Soleus muscle cellular density, *p=0.0003 (TG) and P<0.0001 (TLG) vs CG (B) TA muscle cellular density *p=0.0045 (TG) and P<0.0001 (TLG) vs CG; **p=0.00074 vs TG. (Mean±SD).

Muscle fiber density analysis revealed that the endurance training produced a significant decrease in the soleus [TG (p=0.0003, CI=1.264 to 4.150); TLG p<0.0001, CI=2.474 to 5.093)] and TA [TG (p=0.0045, CI=1.492 to 8.309); TLG (p<0.0001, CI=5.884 to 13.12) fiber density compared to the CG (Figures 4A and B). Furthermore, the TLG showed a significant reduction in TA fiber density compared to the TG (p=0.0074, CI=1.191 to 8.008; Figure 4B).

### Immunohistochemistry

#### Myogenin expression

**Figure 5.**
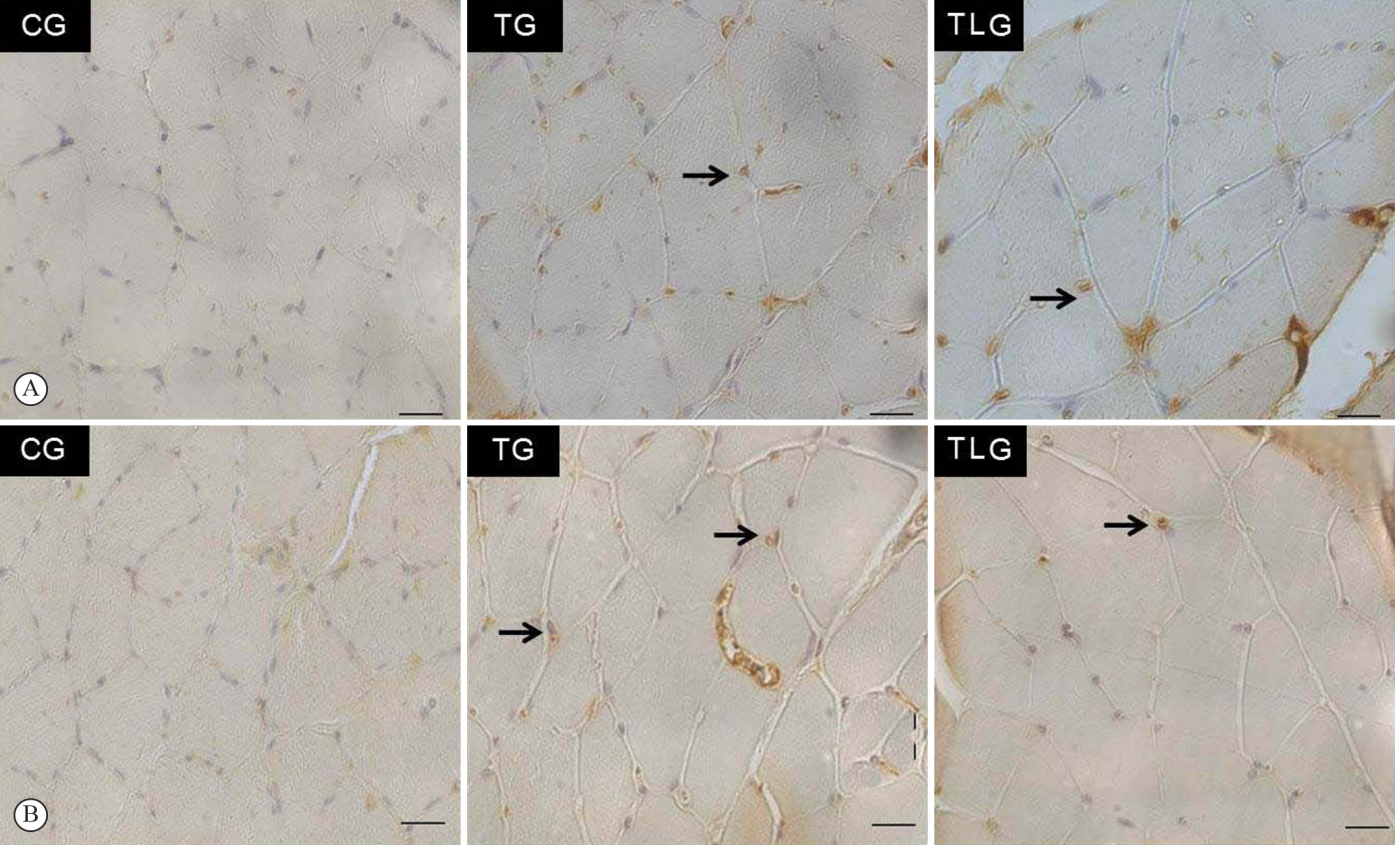
Representative sections of myogenin immunohistochemistry. (A) Soleus muscle; (B) Tibialis Anterior. Immunolabeled muscle cell (arrow); Sedentary control group (CG); trained group (TG); trained and laser irradiated group (TLG). Scale bar 20 µm.

Figures 5A and 5B showed myogenin immunohistochemistry of soleus and TA muscle. Myogenin expression was observed in the nucleus of the muscle cells for both muscles in the trained animals.

In the soleus muscle, no immunoexpression of myogenin was observed in the CG (Figure 5A). Similar immunostaining was observed in the TG and TLG.

In the TA, no immunomarked nucleus was observed in the CG (Figure 5B). Moreover, a higher myogenin expression was observed in the TG compared to the TLG.

## Discussion

This study aimed to evaluate the effects of LLLT in conjunction with an endurance training protocol on biochemical markers and morphology of skeletal muscle in rats. The main findings revealed that the exercise-trained rats showed a significant increase in the speed of running compared to the CG but there was no difference in the speed between the TG and the TLG. In addition, LLLT produced a decrease in lactate levels, an increase in TA fiber CSA and a decrease in TA fiber density. Furthermore, laser therapy produced a decrease in myogenin expression in the TA of trained animals.

Endurance exercise training has been shown to induce a series of physiological and biochemical adaptations in skeletal muscle, which is related to improved muscle efficiency and better physical performance^23^^,^ 24. In the present study, LLLT did not offer any extra stimulus to increase the speed of the exercise rats during the test.

Blood lactate concentration is one of the most common parameters used to evaluate physical capacity during performance testing in athletes, and it is used as an effective variable to determine muscle recovery after exercise^25^. Increased levels of serum lactate are associated with intracellular acidification of muscle fibers, which contributes to muscle fatigue^26^. In the current study, a lower lactate concentration was found in the TLG group at the end of the experiment, indicating that LLLT was able to optimize lactate removal, favoring metabolic recovery after exercise. These findings corroborate those of De Marchi et al.^27^, who demonstrated that laser irradiation applied before a progressive-intensity running exercise program decreased CK and lactate dehydrogenase (LDH) enzyme concentration, protecting skeletal muscle against exercise-induced damage and improving muscle performance. In addition, Patrocinio et al.^28^ demonstrated that laser irradiation applied after a resistance training exercise program reduced lactate levels at rest and improved muscle fiber morphology, increasing muscle performance during a resistance exercise protocol. The beneficial effects of laser therapy on lactate removal may be related to the increase in microcirculation, the stimulation of mitochondrial activity, and the enhancement of ATP synthesis produced by LLLT^29^.

Physical exercise promotes an adaptive response from the skeletal muscle that involves a series of molecular signaling pathways that lead to increased expression of contractile proteins and an eventual increase in muscle size and strength^23^. In this study, morphometric analysis showed that an endurance training protocol increased soleus and TA fiber CSA and decreased fiber density in both muscles. Moreover, LLLT produced a greater increase in TA fiber CSA and a significant reduction in TA fiber density compared to the trained groups only. Some authors showed that LLLT is capable of enlarging muscle fiber diameter in different experimental models, and this effect has been attributed to the stimulatory potential of LLLT, which can produce neoangiogenesis, increase muscle satellite cell proliferation, and upregulate the expression of growth factors^30^^,^ 31. Thus, the results of the present study may be explained by the positive action of LLLT on the modulation of the expression of myogenic transcription factors and consequent satellite cell proliferation that could have contributed to the increase in fiber CSA and consequently the decrease in fiber density.

Furthermore, myogenin expression was noticed in the TA and soleus in both trained groups and was not observed in the CG. Interestingly, the combination of endurance training and LLLT produced a decrease in myogenin expression in the TA muscle. Some studies have recently demonstrated a direct relationship between expression of myogenic regulatory factors and exercise performance^31^^,^ 32. Flynn et al.^33^ found improved performance during high- and low-intensity treadmill running in myogenin-deleted mice compared to controls. The authors suggest that the enhanced exercise capacity in the absence of myogenin is related to the improved oxidative and glycolytic metabolism. Moreover, other studies found that the deletion of myogenin in adult mice enhanced their exercise endurance by altering their skeletal muscle metabolism demonstrated by increased oxygen consumption and alterations in blood metabolite concentrations during exercise^26^. Thus, the lower expression of myogenin in the laser-trained group in this study led us to infer that the LLLT may optimize the oxidative metabolism, improving the efficiency during an endurance-training program.

Nevertheless, the outcomes of the current study highlighted the effectiveness of the laser parameters used in the stimulation of the exercised muscular tissue. The parameters chosen in this study were based on the study by Patrocinio et al.^28^, who investigated the action of 808 nm laser (infrared) applied after a resistance-exercise protocol and demonstrated a positive effect of this irradiation in increasing muscle performance. It is well known that there is no consensus in the literature on the ideal laser regime to be used in different clinical conditions. It is possible to find studies investigating the effects of both red and infrared lasers in muscle performance after an exercise program, both in humans and rats^12^^,^ 13 ^,^ 16. Moreover, different values of energies were used by different authors (from 0.1 J per point until 60 J per point); however, the suite of parameters to be used in clinical therapies does warrant further investigation. In addition, different approaches of irradiation have been used, such as LEDTs. Leal et al.^19^ compared the effect of LLLT and LEDT in lower limb muscle before heavy exercise. The results demonstrated that neither performance nor blood lactate levels were significantly affected by pre-exercise LEDT or LLLT. However, the suite of parameters to be used in clinical therapies does warrant further investigation.

As this study was limited to the analysis of biochemical markers and muscle morphology, the investigation of cell and molecular pathways involved in the positive action of LLLT in exercised rats remains to be provided. Further investigations are required to evaluate possible response mechanisms that may explain the positive outcomes obtained when examining LLLT combined with an endurance training protocol. Additionally, the present study allowed us to obtain preliminary data about the potential of LLLT in stimulating muscular tissue in exercised rats, which supports the evidence for the developing of clinical trials in different populations such as elderly people and athletes. Such future studies will undoubtedly contribute to a better understanding of the safety and effectiveness of LLLT in sport medicine.

## Conclusion

The results of the current work indicate that LLLT decreased lactate concentration at rest, improved muscle fiber morphology, and decreased myogenin expression in the trained rats, which may have contributed to the optimization of the physical recovery in chronic exercised rats compared to non-irradiated animals. Consequently, these data highlight the potential of LLLT as an alternative to stimulate muscle metabolism during physical exercise. Further research involving other LLLT parameters and clinical works are required in order to establish an ideal protocol of irradiation.

## References

[1] Renno AC, McDonnell PA, Parizotto NA, Laakso EL (2007). The effects of laser irradiation on osteoblast and osteosarcoma cell
proliferation and differentiation in vitro. Photomed Laser Surg..

[2] Bossini PS, Fangel R, Habenschus RM, Renno AC, Benze B, Zuanon JA (2009). Low-level laser therapy (670 nm) on viability of random skin flap in
rats. Lasers Med Sci..

[3] Assis L, Moretti AI, Abrahão TB, Souza HP, Hamblin MR, Parizotto NA (2013). Low-level laser therapy (808 nm) contributes to muscle regeneration
and prevents fibrosis in rat tibialis anterior muscle after
cryolesion. Lasers Med Sci..

[4] Chung H, Dai T, Sharma SK, Huang YY, Carroll JD, Hamblin MR (2012). The nuts and bolts of low-level laser (light) therapy. Ann Biomed Eng..

[5] Karu TI, Kolyakov SF (2005). Exact action spectra for cellular responses relevant to
phototherapy. Photomed Laser Surg..

[6] Vieira WH, Ferraresi C, Perez SE, Baldissera V, Parizotto NA (2012). Effects of low-level laser therapy (808 nm) on isokinetic muscle
performance of young women submitted to endurance training: a randomized
controlled clinical trial. Lasers Med Sci..

[7] Manteĭfel' VM, Karu TI (2004). Increase in the number of contacts of endoplasmic reticulum with
mitochondria and plasma membrane in yeast cells stimulated to division with He-Ne
laser light. Tsitologiia.

[8] Leal EC, Lopes-Martins RA, Frigo L, De Marchi T, Rossi RP, Godoi V (2010). Effects of low-level laser therapy (LLLT) in the development of
exercise-induced skeletal muscle fatigue and changes in biochemical markers
related to postexercise recovery. J Orthop Sports Phys Ther..

[9] Leal EC, Lopes-Martins RA, Vanin AA, Baroni BM, Grosselli D, De Marchi T (2009). Effect of 830 nm low-level laser therapy in exercise-induced skeletal
muscle fatigue in humans. Lasers Med Sci..

[10] Leal EC, Lopes-Martins RA, Baroni BM, De Marchi T, Taufer D, Manfro DS (2009). Effect of 830 nm low-level laser therapy applied before high-intensity
exercises on skeletal muscle recovery in athletes. Lasers Med Sci..

[11] Ferraresi C, Hamblin MR, Parizotto NA (2012). Low-level laser (light) therapy (LLLT) on muscle tissue: performance,
fatigue and repair benefited by the power of light. Photonics Lasers Med..

[12] Leal-Junior EC, Vanin AA, Miranda EF, Carvalho PT, Dal Corso S, Bjordal JM (2015). Effect of phototherapy (low-level laser therapy and light-emitting
diode therapy) on exercise performance and markers of exercise recovery: a
systematic review with meta-analysis. Lasers Med Sci..

[13] Borsa PA, Larkin KA, True JM (2013). Does phototherapy enhance skeletal muscle contractile function and
postexercise recovery? A systematic review. J Athl Train..

[14] Toma RL, Tucci HT, Antunes HK, Pedroni CR, Oliveira AS, Buck I (2013). Effect of 808 nm low-level laser therapy in exercise-induced skeletal
muscle fatigue in elderly women. Lasers Med Sci..

[15] Reis FA, Silva BA, Laraia EM, Melo RM, Silva PH, Leal-Junior EC (2014). Effects of pre- or post-exercise low-level laser therapy (830 nm) on
skeletal muscle fatigue and biochemical markers of recovery in humans:
double-blind placebo-controlled trial. Photomed Laser Surg..

[16] Sussai DA, Carvalho PT, Dourado DM, Belchior AC, Reis FA, Pereira DM (2010). Low-level laser therapy attenuates creatine kinase levels and
apoptosis during forced swimming in rats. Lasers Med Sci..

[17] Almeida P, Lopes-Martins RA, De Marchi T, Tomazoni SS, Albertini R, Corrêa JC (2012). Red (660 nm)and infrared (830 nm) low-level laser therapy in skeletal
muscle fatigue in humans: what is better?. Lasers Med Sci..

[18] Ferraresi C, Oliveira TB, Zafalon LO, Reiff RBM, Baldissera V, Perez SEA (2011). Effects of low level laser therapy (808 nm) on physical strength
training in humans. Lasers Med Sci..

[19] Leal EC, Godoi V, Mancalossi JL, Rossi RP, De Marchi T, Parente M (2011). Comparison between cold water immersion therapy (CWIT) and light
emitting diode therapy (LEDT) in short-term skeletal muscle recovery after
high-intensity exercise in athletes--preliminary results. Lasers Med Sci..

[20] Baroni BM, Leal EC Jr, Geremia JM, Diefenthaeler F, Vaz MA (2010). Effect of light-emitting diodes therapy (LEDT) on knee extensor muscle
fatigue. Photomed Laser Surg.

[21] Calegari VC, Abrantes JL, Silveira LR, Paula FM, Costa JM, Rafacho A (2012). Endurance training stimulates growth and survival pathways and the
redox balance in rat pancreatic islets. J Appl Physiol (1985).

[22] Mandarim-de-Lacerda CA, Fernandes-Santos C, Aguila MB (2010). Image analysis and quantitative morphology. Methods Mol Biol..

[23] Mahoney DJ, Tarnopolsky MA (2005). Understanding skeletal muscle adaptation to exercise training in
humans: contributions from microarray studies. Phys Med Rehabil Clin N Am..

[24] Yan Z, Lira VA, Greene NP (2012). Exercise training-induced regulation of mitochondrial
quality. Exerc Sport Sci Rev..

[25] Nielsen J, Schrøder HD, Rix CG, Ortenblad N (2009). Distinct effects of subcellular glycogen localization on tetanic
relaxation time and endurance in mechanically skinned rat skeletal muscle
fibres. J Physiol..

[26] Urtado CB, Pereira GB, Urtado MB, Carvalho EB, Leite GS, Donatto FF (2011). Resistance training associated with the administration of
anabolic-androgenic steroids improves insulin sensitivity in ovariectomized
rats. Diabetes Metab Syndr Obes..

[27] De Marchi T, Leal EC, Bortoli C, Tomazoni SS, Lopes-Martins RA, Salvador M (2012). Low-level laser therapy (LLLT) in human progressive-intensity running:
effects on exercise performance, skeletal muscle status, and oxidative
stress. Lasers Med Sci..

[28] Patrocinio T, Sardim AC, Assis L, Fernandes KR, Rodrigues N, Renno AC (2013). Effect of low-level laser therapy (808 nm) in skeletal muscle after
resistance exercise training in rats. Photomed Laser Surg..

[29] Xu X, Zhao X, Liu TC, Pan H (2008). Low-intensity laser irradiation improves the mitochondrial dysfunction
of C2C12 induced by electrical stimulation. Photomed Laser Surg..

[30] Shefer G, Barash I, Oron U, Halevy O (2003). Low-energy laser irradiation enhances de novo protein synthesis via
its effects on translation-regulatory proteins in skeletal muscle
myoblasts. Biochim Biophys Acta..

[31] Nakano J, Kataoka H, Sakamoto J, Origuchi T, Okita M, Yoshimura T (2009). Low-level laser irradiation promotes the recovery of atrophied
gastrocnemius skeletal muscle in rats. Exp Physiol..

[32] Meadows E, Flynn JM, Klein WH (2011). Myogenin regulates exercise capacity but is dispensable for skeletal
muscle regeneration in adult mdx mice. PLoS One..

[33] Flynn JM, Meadows E, Fiorotto M, Klein WH (2010). Myogenin regulates exercise capacity and skeletal muscle metabolism in
the adult mouse. PLoS One.

